# Demands on Health Information and Clinical Practice Guidelines for Patients from the Perspective of Adults with Mental Illness and Family Members: A Qualitative Study with In-Depth Interviews

**DOI:** 10.3390/ijerph192114262

**Published:** 2022-11-01

**Authors:** Katja Schladitz, Elena C. Weitzel, Margrit Löbner, Bettina Soltmann, Frank Jessen, Jochen Schmitt, Andrea Pfennig, Steffi G. Riedel-Heller, Uta Gühne

**Affiliations:** 1 Institute of Social Medicine, Occupational Health and Public Health (ISAP), Medical Faculty, University of Leipzig, 04103 Leipzig, Germany; 2 Institute of Clinical Psychology and Psychotherapy, Medizinische Fakultät Carl Gustav Carus, TU Dresden, 01062 Dresden, Germany; 3 Department of Psychiatry and Psychotherapy, Faculty of Medicine, University Hospital Cologne, University of Cologne, 50937 Köln, Germany; 4 Center for Evidence-Based Healthcare (ZEGV), Medizinische Fakultät Carl Gustav Carus, TU Dresden, 01069 Dresden, Germany

**Keywords:** patient guideline, patient health information, health literacy, mental illness, mental health

## Abstract

(1) Background: “Patient health information” promote health literacy. “Patient guidelines” as a sub group reflect the current evidence about illnesses and treatment options adapted to the needs of laypersons. Little is known about factors promoting and hindering their use by people affected by mental illness and their relatives. (2) Methods: Telephone interviews (*N* = 15; *n* = 4 adults affected by mental illness, *n* = 5 relatives, *n* = 6 both applicable) were conducted according to the *Sørensen* model of health literacy. Data were recorded, transcribed and content-analyzed following *Mayring*. (3) Results: Health information is used regularly by individuals affected by mental illness and their relatives, but “patient guidelines” are largely unknown. Yet, there is a great willingness to use them. Main barriers are a lack of statistical knowledge, the complexity of health-related topics and cognitive impairment sometimes accompanying mental illnesses. Target group-oriented adaptation as well as transparent and even-handed presentation of (dis-)advantages of treatment options can increase trust. (4) Conclusions: Health information and guidelines can help affected persons and relatives to make treatment decisions by conveying unbiased, up-to-date knowledge. Target group-specific adaptations should be made for psychiatric illnesses and features specific to mental illnesses compared to physical illnesses should be included. Clinical practice guidelines must be distributed more widely to increase their impact.

## 1. Introduction

Mental illnesses affect a significant portion of the global population: between one-quarter and one-third of the adult population will meet the diagnostic criteria for a mental disorder across their lifetime (varying between 25.9–32.6%, pooled prevalence 29.2%) [1]. They are one of the main contributors to the global burden of disease (estimated as accounting for 7% of disability-adjusted life years) [2]. Yet there is a global gap in treatment which is partially accounted by low treatment seeking, treatment availability, and insurance coverage of mental illnesses, factors which deteriorate clinical, social, and socioeconomic outcomes [3,4,5,6,7]. Furthermore, the treatment gap is associated with sociodemographic and socioeconomic characteristics [4,6], as well as care-related factors, such as difficulties in navigating complex health care systems [8] and low acceptance of mental treatment services [9]. Evidence suggests that those who perceive a need for mental health care are more likely to meet that need and seek treatment for mental health problems [10].

The ability of a person to recognize when there is a medical condition that requires treatment and to know how to seek health care is defined as health literacy. According to Sørensen et al. [11], health literacy is a key prerequisite for navigating through the health care system and making decisions that have a positive impact on one’s own health. It is comprised of four crucial dimensions: (1) access, (2) understanding, (3) appraisal and (4) adequate application of health information. Sufficient development of these dimensions is a prerequisite to acquiring the knowledge and skills to promote one’s own medical care when ill, to reduce the risk of disease and to promote one’s own state of health throughout life course. The European Union (EU) has identified health literacy as a key determinant which needs to be increased in order to improve the health status of the population and the effectiveness of health care. The results from the European Health Literacy Survey (HLS-EU) showed that 47.6% have low health literacy (12.4% were rated “inadequate” and 35.2% were “problematic” in terms of literacy) [12]. Current results from the representative Health Literacy Survey Germany 2 (HLS-GER 2) reveal even more significant deficits: more than half the population (58.8%) have low health literacy (28.4% “inadequate” and 30.4% “problematic” literacy) [13]. There is empirical evidence that people with mental illnesses have even lower health literacy than the general population [14].

Mental health literacy includes information about prevention as well as how to recognize and manage mental illnesses. This includes understanding how to obtain and maintain positive mental health, understanding mental illnesses and their treatment, decreasing stigma and enhancing help-seeking efficacy [15,16]. Higher mental health literacy was identified as a predictor of higher use of mental help services and, therefore, positively influences clinical and social outcomes of people affected by mental illnesses [17]. Also, there is evidence that higher mental health literacy is significantly associated with increased formal help-seeking from health professionals, as well as informal help-seeking from family members and friends [18]. There is increasing consensus that family members of individuals affected by mental illness should be included in therapy decisions [19,20,21].

Evidence-based health information materials, which provide an unbiased and trustworthy description of the current state of medical knowledge [22], and decision aids are important resources for raising health literacy and enable individuals with illnesses to make informed decisions concerning their therapy [23]. They enhance knowledge about standard and alternative treatment options including benefits and risks of interventions, give people affected the feeling of being better informed and clearer about their values [24]. Individuals affected by illness have a right to access evidence-based health information that can be understood by laypersons [25,26]. Yet, a recent study [27] showed that most health information had low readability and required a higher literacy than recommended. To facilitate the acquisition of knowledge, evidence-based health information should be adequately tailored to the target group and its informational needs [28]. 

Clinical practice guidelines (CPGs) are one specific type of evidence-based health information. These statements are systematically developed by a multidisciplinary panel of experts and are intended to optimize health care quality. They synthesize current scientific evidence about an illness based on a systematic review of the literature and provide ratings of the quality of evidence and the strength of recommendations (with assessing the benefits and harms of alternative care options). CPGs aim to assist clinicians, individuals affected by an illness and their family members in making decisions about appropriate health care considering specific circumstances and individual needs [28,29,30]. Typically, they address healthcare providers but there is increasing interest in versions for the public (individuals affected by a mental or physical illness and their families) [31,32,33]. Research literature shows a low level of awareness of CPGs in general [31,34] and especially among people affected by mental illnesses and their family members [35]. For the psychiatric field, in particular, little is known about the barriers and facilitating conditions in the dissemination and implementation of evidence-based health information including CPGs, as most research has focused on physical diseases [36]. 

The aim of this qualitative study was to inquire about previous experiences with psychiatric health information from individuals affected by mental illness and their families and to identify needs and specific requirements for evidence-based health information, especially CPGs. In particular, the focus was on accessing, understanding, appraising and applying health information, considering barriers and facilitating factors as well as potential differences regarding mental and physical illnesses. These findings are intended to improve the target group-oriented design of CPGs to promote the health literacy of individuals affected by mental illness and their family members in the future.

## 2. Materials and Methods

### 2.1. Study Design

This study uses an exploratory qualitative approach employing individual telephone interviews with adults affected by mental illness and family members and is reported according to the Standards for the Presentation of Qualitative Research Results (SRQR) [37]. The semi-structured interview guide was developed in a qualitative research workshop, in which questions were derived deductively based on the Sørensen et al., integrated model of health literacy [11] and on current literature searches. The interview guide applies the questioning technique for problem-centered interviews as outlined by Witzel [38]. This approach is particularly appropriate for exploring subjective experiences and attitudes towards the topic of patient health information. The structure of the interview allows a consistent orientation towards the subject and the interview process, while at the same time being flexible and open for emerging topics. 

### 2.2. Participants and Settings

**Figure 1:**
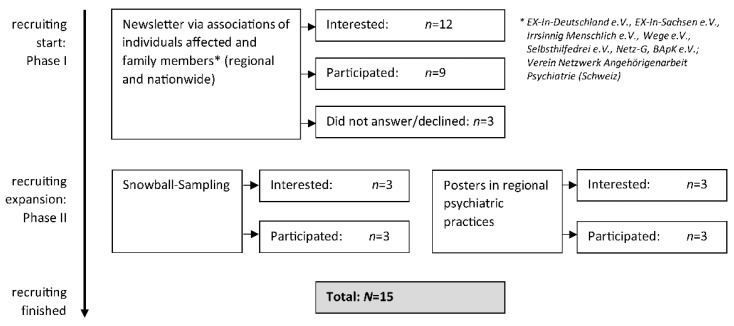
Flowchart of recruitment process.

Initially, participants were recruited via self-help associations for people affected by mental illness and family members: contact persons were asked to spread the study invitation via newsletter. After one month, we revised our recruitment strategy due to low recruitment success following approved strategies by Röhr et al. [39] and adopted additional activities (see Figure 1 for details). Since it was particularly difficult to recruit family members, we also asked individuals affected by mental illness if there was a friend or family member who may also wanted to give us an interview (therefore using purposive and snowball sampling in the participants’ social environment). We also hung posters in regional psychiatric practices. 

To be included in the study, prospective participants had to be affected by a mental illness or to be related with a person affected (not restricted on proximity of kinship), as well as to be fluent in German. Interested participants were asked to contact the study organization by e-mail or telephone. They were sent detailed study information, a consent form, an account data form and a questionnaire with sociodemographic questions and questions about sources of health information, by post in advance. 15 prospective participants who were either personally asked for participation or made contact on own initiative fulfilled the inclusion criteria and consented to participate. One person was willing to participate, but had to but had to cancel due to scheduling difficulties.

### 2.3. Data Collection

In-depth telephone interviews (*N* = 15) were conducted from August–November 2021 by a psychologist and research assistant with expertise in qualitative research and by a trained student in the master’s program in psychology under supervision. The semi-structured interview guide began with two open-ended questions: “What role does the topic of health play in your everyday life?” and “To what extent do you engage with health information in your daily life?”. The open-ended nature of the questions encouraged participants to spontaneously describe their experiences. More specific questions followed on their experiences with health information (especially access, understanding, appraisal, and application, considering barriers and facilitating factors), current use, needs regarding health information including CPGs, access paths and willingness to use CPGs. Specific needs of individuals affected by mental illness and family members, as well as potential differences between mental and physical illnesses regarding health information, were addressed. Duration of interview sessions ranged from 25–128 min (*M* = 61:48 min). Data were collected and analyzed simultaneously. Theoretical saturation according to Glaser & Strass [40] was discussed between the two researchers as well as in the complete research team and considered as achieved when no new topics were raised by the participants. According to our evaluation, further interviews and analyses would not provide any new insights. Data collection was therefore stopped after 15 interviews.

### 2.4. Data Analysis

The interviews were audio-recorded with the participant’s permission, fully transcribed either in the study center by trained students in the master’s program in psychology under supervision or externally by an established transcription service according to extended transcription rules for scientific topics (literal with minor language correction as well as adaptation of dialects to the German standard language). All transcripts were checked and proofread by students and psychological research assistants.

The interviews were content-analyzed using MAXQDA 2018. The derivation of the coding scheme followed a combined deductive and inductive approach (deductive from the model-based interview guide, inductive from the audio material) according to the protocol for qualitative analysis by Mayring and Fenzl [41] (deductive development of the coding system, revision, coding of the transcripts with addition of inductively derived codes, determination of the final coding system, back-checking on the data material). Trained psychological research assistants with experience in qualitative research independently coded and revised both interviews. They performed a preliminary analysis, then reviewed and compared the initial coding and discussed codes that could not be clearly assigned. These iterations of analysis, comparison and refinement were repeated frequently which led to a more representative coding scheme with main categories, sub categories and sub themes. At longer intervals, the coding system was discussed in the interprofessional research team which also consented the final version. Consensus and methodological stringency were established by mutual agreement during the intermediate steps and in a final group discussion. 

### 2.5. Ethical Considerations and Data Protection

The project was approved by the Ethics Committee of the Medical Faculty of the University of Leipzig (243/21-ek on 8 June 2021). Participants were informed about the purpose of the study, reporting of study results, and interview recordings. We obtained written informed consent from all participants before data collection. In addition, it was explained that participants could withdraw from the study at any time without any harm if they wished. All participants received an incentive of €40. 

Each participant was assigned a neutral “project ID” that does not contain any information from which the identity of the participant can be inferred. The audio files were stored in password-protected electronic form on a secure drive and were only accessible for the purpose of study evaluation by authorized project members. The transcripts had been anonymized (personal or geographical names and other information that could allow conclusions about the identity of participants had been removed). Data will be saved for ten years and then deleted, according to the European Union’s (EU) and German General Data Protection Regulation.

## 3. Results

Results are based on 15 qualitative interviews with adults affected by mental illness and family members. Sociodemographic data are listed in Table 1. Participants were between 30–74 years old, *n* = 12 were female. All participants finished at least the secondary school (*n* = 4) or high school (*n* = 11) and *n* = 6 acquired an academic degree. Six participants were affected by mental illness themselves as well as family member of an affected individual, four were affected by mental illness only themselves, and five were solely family member of an affected person. Concerning psychiatric diagnosis (multiple answers were allowed), affective and anxiety disorders were mentioned most frequently (unipolar depression: *n* = 10, anxiety disorder: *n* = 4; bipolar disorder *n* = 3). The reported illness persisted between 2–27 years. 

**Figure 2:**
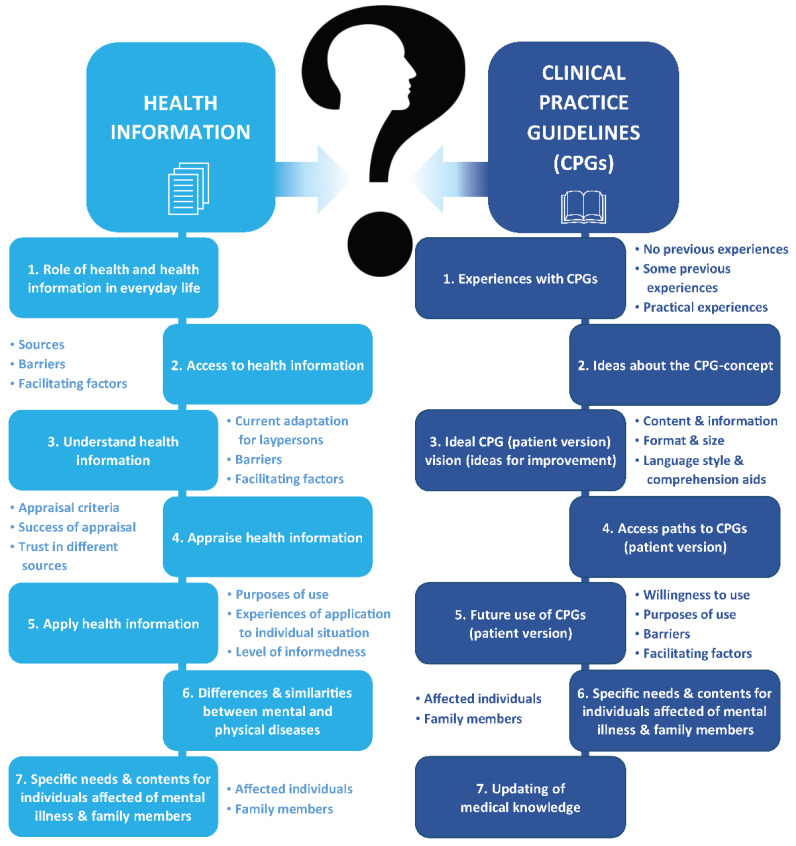
Thematic map of main categories, sub categories and (if applicable) sub themes identified in qualitative semi-structured interviews on patient health information and clinical practice guidelines with *N* = 15 individuals affected by mental illness and family members of affected adults.

Participants were asked about their experiences and needs regarding patient health information and CPGs. Results are subsequently presented separately for the two main categories (1) health information and (2) CPG (see Figure 2 for all categories and themes). 

### 3.1. Main Category 1: Health Information

#### 3.1.1. Role of Health and Health Information in Everyday Life

All participants reported that health topics played a significant role in everyday life for purposes of prevention (e.g., nutrition, exercise, stress management, psychological well-being) as well as treatment of their own mental and physical illnesses and those of family members.

*“Yes, a big role, if not the most important, because if you don’t have your health you’re in a bad way. A healthy lifestyle and way of life is the be-all and end-all, so that you can feel well and go about your daily business, your hobbies and so on, and be there for your family […].”*

(B08)

They obtained health information both purposefully (e.g., by doing specific research or attending lectures), and randomly (e.g., in conversations or media). For some participants, the topic of health information played a role not only privately, but also professionally or in their voluntary activities in a health-related field.

#### 3.1.2. Access to Health Information

Participants mentioned the internet (primarily websites, less frequently forums), magazines, books and brochures and their healthcare providers (e.g., medical doctors and psychotherapists) as main *sources for accessing health information* (Sub themes of sub categories in Section 3 are presented in *italic*) which is congruent with results from the short questionnaire filled out by the participants (internet 93.3%, books, fact sheets and brochures 53.3% each, talks with medical doctors 40%—multiple answers were allowed). Less frequently, access was also provided by:

- Daily newspaper
- Associations (e.g., mutual support and self-help, peer counseling) and professional societies
- Health insurance companies (e.g., prevention programs, newsletter)
- Flyer in waiting rooms
- Social environment (e.g., family members, friends, colleagues, persons affected by similar illnesses)
- Documentaries on television, movies, radio programs, podcasts
- Advertisement in public streets and transport
- Academic studies and further education (e.g., medicine, psychology, social work), “health information” days in schools, lay lectures on health topics.

*“On the internet […] a quick Google search if I’ve got a niggle and that sort of thing as a source of information. […] I like to pick up health magazines at the pharmacy, sure. But if there are particular issues that affect me, or affect me as a family member particularly now that my mother is ill, then I’ll find literature and books and I’ll look for advice on recommended books at the [author’s note: self-help] association.”*

(B08)

These *barriers to access* relevant health information were mentioned:

- Primarily, lack of knowledge about sources for specific information (especially about institutions, associations and consultancy services)
- Information overload (especially when searching online), resulting in difficulties filtering out optimal information for the individual situation from the high number of results
- Difficulties successfully performing a search when very specific information is needed (e.g., on a more specific topic and not on the most common therapy option)
- When a certain prior knowledge was needed as a prerequisite for a successful search (e.g., specialist terms, psychiatric diagnosis)
- Lack of clarity of available support structures as well as bureaucratic barriers (e.g., when applying for financial or socio-medical support)
- Convoluted web portal pages
- Long waiting times for therapy appointments
- Lack of drive (e.g., in acute phases of a mental illness)
- Stigmatization of mental illnesses inhibited seeking help.

*“Once you find the right place, you’ll find really good information on certain sites or about certain topics. Well presented, too. I think the real problem is getting there. Or that there’s just too much information out there that’s not necessarily useful.”*

(B07)

Mentioned *facilitating factors* for finding relevant health information:

- Offers tailored to specific target group and its needs (e.g., lectures for family members, psychoeducation in day or inpatient clinics or rehabilitation)
- Enabling a targeted internet search via everyday language terms and symptom descriptions
- Provision of information free of charge
- Provision of information in everyday contexts (e.g., daily newspaper or advertisements in public spaces)
- Central platforms or well-known reputable websites as starting points for an internet search
- Receive information automatically without having to act yourself or to ask (e.g., via physicians, socio-medical or psycho-social supporters or health insurance companies)
- Feeling addressed by the presentation of health information (e.g., because of design or appeal).

*“When I was first confronted with the illness in 1999 and my son was admitted to hospital, they organized a seminar for family members for the very first time. We were told about the individual illnesses and that happened ten times over the course of an evening, and that was really good.”*

(B11)

#### 3.1.3. Understand Health Information

Currently, participants perceived health information (websites, flyer, brochures, books, and newspapers) as difficult to understand. The language and writing style required too much prior knowledge. This was especially a problem when searching on a new topic (e.g., after a new diagnosis is received). When people became better informed, their understanding developed. Participants who have been dealing with health information on mental illnesses for many years perceived improvement over time, especially when individuals affected with mental illnesses were involved in elaborating content and wording. Phrasing was described as more comprehensible today as compared with the past.

*“It varies, of course. I’ve seen many texts that I find difficult to understand in the sense that they’ve used medical terms or simply tried to sound fancy, especially when talking about the psyche, so that you always need a certain amount of background knowledge or a certain IQ level, so to speak […].”*

(B04)

Furthermore, specific *factors that complicated comprehension* were mentioned. First, the complexity of health issues (e.g., large scope and richness of detail) and legal regulations (e.g., when seeking financial support and applying for socio-medical assistance) were difficult to understand. A second group of comprehension-impairing factors were related to the individual. Each person had different capabilities for dealing with these barriers, so these factors can be regarded as additional barriers to comprehension (e.g., statistical knowledge as a prerequisite to understanding study results, reduced cognitive performance and concentration in acute phases of a mental illness, as a symptom of a mental illness, or as a side effect of some medications).

*“A bigger problem, as I see it, are statistical estimates and results, for instance. […] Such complex matters just aren’t so easy to explain. And you probably need deeper knowledge of a topic to be able to evaluate study results or to even understand on what basis or how study results are achieved, and what it means to have a result that clearly leads to a certain result in this case.”*

(B07)

*“[…] when affected people read it […] they are mostly going through a phase where they are not doing well at all. They are not being receptive. I’ve experienced it myself. I also noticed in the hospital, in psychoeducation, that the first few times there just went completely past me. I couldn’t absorb it at all because I wasn’t well enough. And it’s exactly the same if you try to read something in that state. Not a lot of it will stick, unfortunately.”*

(B01)

Participants identified the following factors that could facilitate understanding of health information:

- Simpler wording, complemented with a version or summary following easy-to-read rules
- Explanation of specialist terms
- Inclusive and stigma-free language style, involving individuals affected by mental illness in the production and revision of health information
- Design elements to promote comprehension (e.g., creating a structure with headings, lists and paragraphs; visualization with graphics, diagrams, pictures and tables)
- Multiple versions: “introductory version” for people with little prior knowledge, “advanced version” for more experienced people
- Inclusion of field reports and case studies
- Additional multi-media elements (e.g., print and digital products, audio and video material).

*“[…] then again, the easy-to-read language is too easy for some people […] It’s important for people with psychiatric diagnoses that there is something between the lofty language or jargon, whether bureaucratic or medical, and the easy-to-read language. There has got to be something in between. And there have been attempts at that, I think. But it shouldn’t come across as didactic, I mean it must be self-explanatory. The vocabulary should be easily understandable.”*

(B03)

#### 3.1.4. Appraise Health Information

Participants considered the source of health information to be the most important *criterion* for its appraisal. They classified scientific or medical publishers as reputable. Some also regarded the experiences of individuals affected of mental illnesses as trustworthy.

*“I think it depends on the source. If I trust the source, then I’m more likely to trust the information too. […] I trust things more if they are presented to me as a possibility and an option and maybe as predominantly good experiences or recommendations, but not as the ultimate truth.”*

(B10)

Participants also expected health information to be based on current scientific knowledge. Furthermore, reliable health information should represent all relevant points of view and include advantages as well as disadvantages of each therapeutic option. It should be transparent how the authors derived their conclusions. Comprehensibility as well as applicability and suitability to individual needs were also relevant.

*“The balance between pros and cons, limitations, possibilities, whatever they may be. Diversity, that is, that they’re not just trying to make a specific point but instead you can see that someone’s considered the big picture, not just the topic at hand or whatever might be in their interest.”*

(B10)

Participants said that they were now *able to evaluate* health information well to very well. However, this was difficult at the beginning, when a specific health topic or diagnosis was new to them—the more knowledge and personal experience they gained, the more successful their appraisal. There was no difference between mental and physical illnesses.

*“I do my best, yes. In books, the sources are mostly stated properly. On the internet, let’s just say, I pay close attention to it, and if it’s a well-founded text, then the sources are usually mentioned. I’d like to think that I’m well-informed these days, if I make the effort. You have to take the time for it, though.”*

(B15)

*Trust* would vary significantly between different sources of health information. Especially when searching online, participants found it difficult to find reliable information. Most internet forums were perceived as untrustworthy. There was a high level of trust in practitioners, health authorities, associations, institutions, foundations, and daily newspapers as well as “educational television” and documentaries. Popular magazines and journals were rated as questionable sources by some participants, since they suspected that publishers had economic interests. Scientific journals, as well as pharmacy and health insurance magazines, on the other hand, were considered to be trustworthy. In the case of books and brochures, a distinction was also made between specialist literature from trustworthy publishers, who were generally regarded as reliable, and individuals with questionable expertise. Acquaintances were also an important source of health information, but their reliability was also be weighed on a case-by-case basis.

*“Well, on the internet you have to look closely at whatever it is you found, what the sources are. There are millions of articles out there and not all of them are trustworthy. Compared to that, a book that I’ve picked out or got from the library is a safer source, a good source indeed.”*

(B08)

#### 3.1.5. Apply Health Information

Participants used health information specifically to

- Raise awareness of illness progression and deterioration of health status
- Acquire psychoeducation (for themselves and for others)
- Optimize prevention behavior
- Search for therapy options
- Develop rules for dealing with ill family members and friends.

*“Yes, of course, if you’ve done some reading, you’re able to, I think, it’s helpful in the sense that you can ask specific questions about a treatment or you can say, look, I’ve heard about this here, could this be an option? It’s useful in that respect.”*

(B15)

Most of the participants reported currently feeling well informed about their individual situation and needs. However, this had taken a long time, had been difficult and had required a lot of personal effort.

*“Yes, by now really well. But that too was a long way. Getting started was slow going, you first had to find out who even is there that can help you along. These days I would say that I’m well-informed, but it wasn’t—. It was a long way to get there.”*

(B15)

Most participants reported difficulties in *applying health information* to their individual situation. The translation into practice and to their specific regional care situation was challenging. When they learned about suitable therapy or psychosocial options, it was difficult to access them (e.g., to find an appropriate provider and to get appointments). They needed a lot of perseverance and time to overcome barriers. It was very difficult to apply for financial or assistance aid (e.g., such as outpatient assisted living) without getting external help. When looking for complementary options (e.g., while waiting for psychotherapeutic treatment or additional to psychotherapy), it was difficult to appraise who worked on a scientific basis and which options were effective. In addition, treatment could be expensive. And in rural regions, therapy availability and accessibility were more likely a problem than in urban areas. As a symptom of mental illness, motivation and drive were often limited and anxiety was sometimes a barrier to getting help. Without external support, it was very difficult to implement health information in everyday life.

*“Often it works. But—. Well, sometimes it’s difficult for family members to judge certain things or to estimate their extent. Or to apply the recommended steps in the first place. That is, to look for a therapy or make an appointment with a specialist, for instance. To even access the appropriate places. I mean, it’s nice that that they say I should see a therapist under certain conditions. But if I can’t reach one or I have to make a large advance payment myself, well that’s a hurdle I’m going to have to clear first.”*

(B07)

Some participants also reported positive experiences of applying health information. For example, they changed their diet or used relaxation and meditation techniques on their own. Health information was used to learn about the mental illness of a family member and to find advice about how to deal with everyday challenges. There were also positive experiences with mutual support and self-help groups for individuals affected by mental illness and their family members. Specifically, the exchange of experiences and the opportunity to network with other affected persons and family members was perceived as very helpful and empowering. Getting active by giving lectures or engaging in psychoeducation and passing on health information also had a positive effect on self-esteem.

*“Yes, very often. When I was sick at home, I did a lot of googling on things like inner restlessness and how to let go. Let go of thoughts I mean. Meditation techniques, relaxation techniques. I did a lot of searching, tried out a lot of things and used them for myself, yes.”*

(B06)

#### 3.1.6. Differences and Similarities between Mental and Physical Diseases

Opposing perceptions were expressed about health information for mental compared to physical illnesses. The majority of participants considered the availability and dissemination of information to be worse in the case of mental illnesses. Psychiatry was seen as a relatively new field of medicine; the etiology and treatment options for psychiatric illnesses were perceived to be less clear compared to physical diseases. In addition, the stigmatization of mental illnesses impeded public discussion of mental disorders. Further because mental illnesses are less visible than many physical illnesses, an artificial division could be drawn. Another perceived difference was that physical disorders could be more easily identified by objective indicators (e.g., blood values or recognizable tissue damage). The most appropriate therapy option depended to a larger extent on individual factors than in the case of physical illnesses. The social environment must also be involved to a greater extent because many mental illnesses can include limited drive and reduced self-awareness of being ill. Some participants, on the other hand, had the impression that they would learn as much or more about mental illness. However, all agreed that there had been a major change in the last decades and that more information about mental illness was now available. There were “fashionable topics” that were very present in public debate and currently there was a lot of information and education about dementia, depression, and burnout.

*“I believe, just because physical illness is naturally easier to isolate and describe, that it’s much easier to gather information and to say precisely, this is what the patient has and these are our options. You can see in the body what the problem is. ‘Mental illness’ in comparison, is a massive field, and you can always only make assumptions, also as a psychiatrist or a therapist, based on what you’re told or what you’ve observed. You can’t really look inside a person. And I think because of that the information on physical things is a lot more specific and you can say that if you’ve got a broken leg, then you’ve got—. If you’ve got coeliac disease, you’ve got to do this or that. But for those with autism, there isn’t a single way that applies to everyone. That’s what I meant before, it’s always only about the individual patient. And what’s good for you varies from person to person. That’s why I find information on physical problems is much easier to present and access.”*

(B09)

#### 3.1.7. Specific Needs and Content for Individuals Affected by Mental Illness and Family Members Regarding Health Information

According to some participants, the phase of illness should be considered: In an acute crisis, the ability to absorb and process information was very reduced, and information should therefore be very simple and limited to what is most necessary in this phase. It would be helpful if family members and friends would be involved in psychoeducation, so that they could pass relevant information to the affected person and to support her. In a stabilized stage, the information could and should be more detailed.

*“I only know that when I was acutely depressed, and I was fairly deeply depressed, I wasn’t able to do or look for anything. I actually could have used someone to take me by the hand or give me a relatively simple text that says: ‘This is what it’s like, these are the typical symptoms. It’s normal, so no need to worry. And these are the treatment options’.”*

(B01)

Authors of health information should emphasize that a mental illness has individual causes, symptoms and patterns for each person and that treatments should be tailored to the needs of the individual person.

*“I have depression, and there are other people who have depression for completely different reasons. What helps can also vary. Some people find medication helpful, others not so much. Others find talking more useful, or just some activities or something. That’s why I think a broad perspective is always important.”*

(B02)

No specific needs were expressed from a family member perspective.

### 3.2. Main Category 2: Clinical Practice Guidelines (CPGs)

#### 3.2.1. Experiences with CPGs

Most participants reported having not heard of CPGs prior to this study. Some were aware that such guidelines existed but had not personally used any. One participant used CPGs in the physical area as part of his medical profession but did not know of any guidelines specific to psychiatric diagnoses. According to the questionnaire, two individuals affected with mental illness (13.3%), one family member and one person who was affected as well as family member (6.7% each) had experience with a patient version of a CPG.

*“No, it may be that I’ve come across it accidentally while searching online, without realizing that it’s a medical guideline, even if I can’t recall it now. But, that someone would have proactively handed one to me, I wouldn’t be aware of it at least.”*

(B09)

According to the participants, CPGs were mostly aimed at specialists and therefore, for their own needs, were too extensive with too much text and too little graphic presentation and comprehension aids. Those participants who were familiar with CPGs for psychiatric diagnoses created specifically for patients and family members reported: the scope and depth of content were appropriate (they understood what it was about, but were not overwhelmed); the language style was easy to read; the text was divided into appropriate paragraphs; and all relevant topics were covered and nothing was missing.

*“I don’t think you can make it shorter. Sure, it was long, but it has to be like this, you can’t condense it any further. It was to the point, without long, convoluted sentences that didn’t pertain to the subject or distracted from the subject. No, it was very good. You could certainly make it a lot longer, but it’s not going to make it more helpful.”*

(B08)

#### 3.2.2. Ideas about the CPG-Concept

Those interview participants who had heard of CPGs before this study described them as a “compendium” or “encyclopedia”, in which one could find a wide range of current scientific findings for an illness and its treatment options. These were based on the consensus of experts and enhanced with links, literature recommendations and addresses. CPGs enabled people to search for answers to questions concerning their illness, to become oriented with treatment options and, in the case of “patient guidelines”, specifically addressed individuals affected by mental illness and their family members.

*“How would I describe a medical guideline? It’s definitely a mixed bag, a diverse and very large collection of information, where you will definitely find what you’re looking for. And this information will also contain further links or tips and literature references. A mixed bag is an appropriate term, or you could also say ‘compendium’.”*

(B08)

After a short explanation CPGs, participants who had never heard of CPGs before were also able to imagine a CPG and to formulate ideas about what they should ideally look like (see the following section).

#### 3.2.3. Ideal CPG (Patient Version) Vision (Ideas for Improvement)

Regarding the optimal design of CPGs, participants described mental health CPGs as comparable to those regarding general health information. Also, participants who had had no previous experience with CPGs formulated very specific ideas about content, format, and scope, as well as language style and comprehension aids. CPGs should contain the following *information*:

- Commonalities of mental illnesses
- Biopsychosocial model
- Etiology of the specific illness
- Clinical symptomatology
- Prevention and treatment options (medication, psychotherapy, psychosocial therapies)
- Mutual support and self-help groups, associations and counseling centers (including local addresses) as a leaflet or online
- Financial support services, socio-medical offers, and legal rights.

*“Firstly, an explanation of the illness itself because, if it’s your first time going through something like it, you have no idea what’s happening to you and you’re doubting yourself. […] it would be great if you could easily find something online […] where it says: ‘OK, these are the symptoms of depression’. Just so you’d know that you don’t have to worry so much and be totally afraid, that it’s normal and also treatable. There is hope, you just have to admit yourself to treatment. Of course, you also have to be open to treatment. […] And then, a relatively simple breakdown of therapy options, what they are good for, what effect they are supposed to have. And the piece of information that options like rehabilitation exist […]”*

(B01)

According to the participants, CPGs should also encourage taking action in one’s own everyday life (beyond the professional care system), while emphasizing that living with a mental illness is possible and can be improved through optimal treatment.

*“To give the affected person or the family, or let’s just say, to give the affected person the feeling that there’s something they can do. And it goes on […] when you notice, I’m doing this well now and I’ve made it back home. What more can I do? And then to give you ideas, oh, here’s another good thing I can do for myself, another way I can support myself in getting better.”*

(B12)

*“That you could get at least halfway back to being well or that you could live a good life as a mentally ill person.”*

(B11)

Regarding *format and size*, participants suggested using a postcard, leaflet or A4 information sheet that would be 1–4 pages in length. It would inform people that there was a CPG for a specific mental illness along with information about where to get it. These “first contact” materials would provide the most important information. The CPG itself should have the form of a brochure since most people would prefer to “hold something in their hand.” There could be a short and a long version, depending on the need and desire for information. In terms of the length of the CPG, an appropriate number of pages varied between 20–50 (one participant even suggested 80–90). DIN A5 or A4 would be an appropriate format. In addition to a printed brochure, there should also be digital versions (a website that could be searched and read on mobile devices, as well as a PDF-file). Smartphone applications could supplement these web pages, but people must also be able to read CPGs on large screens. There should be brochures covering several mental illnesses as an introduction, but also in-depth brochures on specific illnesses.

*“I could imagine a brochure with maybe 15 different descriptions of illnesses. Two to two and a half, two or three pages at most would be good. I think that would be enough to get a good overview.”*

(B12)

Regarding *language style* of CPGs, participants suggested:

- Different versions: (1) standard version with scientific yet comprehensible language, (2) version in easy-to-read language and (3) a version for children
- Avoidance of specialist terms or explanations when necessary for understanding (glossary should be included)
- Optimistic and encouraging as well as inclusive, non-stigmatizing and gender-neutral wording
- Involvement of target group representatives (e.g., by having individuals affected by mental illness check and improve the wording).

*“[…] that you talk about it like you would about any other normal thing and not in a judging tone. […] That the language is kept understandable, as I said, you could perhaps explain medical concepts in simple terms in an aside. That it’s multifaceted […], you could maybe also say, okay, for those who prefer, here’s a link that you can enter on the internet to see an informative video, for instance. For people who are more interested in visual material.”*

(B04)

They suggested following *comprehension aids* in CPGs:

- A summary that provided an overview, followed by more in-depth information
- Text structured in blocks, combined with key points and subheadings
- Case studies and field reports
- Visual elements (e.g., diagrams, pictures, speech boxes, graphical depiction of treatment paths)
- Multimedia elements (e.g., supplementary explanatory videos of 3–10 min).

*“What I think actually makes sense, or what I always really like and sometimes kind of lightens it up is when there are sections here and there with first-person accounts. […] It lightens the whole thing up because it’s something practical.”*

(B02)

#### 3.2.4. Access Paths to CPGs (Patient Version)

According to the participants, CPGs should be easily available. For example, CPGs should be available to those seeking help for mental health problems (e.g., via physician or therapist, flyer or brochure in waiting rooms, health care facilities, self-help groups or via lectures on health topics). As these access routes presupposes that people have already sought help, information about CPGs should additionally be made widely available in public spaces. All places where people go would be appropriate for raising awareness about mental illnesses and reducing stigma. Some examples cited were advertising on the street (e.g., advertising pillars, advertising on public transport), restaurants and cultural institutions, state institutions such as civic or youth welfare offices, libraries, bookstores, supermarkets and funeral homes. City cards, posters and flyers could draw attention to CPGs and provide information about where to obtain them. Media were important (e.g., advertisements in daily newspapers or information in social media). It would be important for CPGs to be available online on trustworthy and well-known sites (e.g., Federal center for health education) and optimized for search engines.

*“They could be available in all sorts of places, flyers maybe or information leaflets. In public libraries or wherever people like to frequent. In opera houses too, for all I care, places of culture like that. Or in bookshops. I wonder who I’m missing though... Where else should they be available? There are cafes with lots of flyers. They could be included. Cinemas as well. Perhaps even places where you wouldn’t necessarily–. Where you wouldn’t expect to be confronted with the topic [author’s note: of mental illnesses]. […] I mean places where you’ll find a great many people and also people of various backgrounds and ages. In order to achieve a really massive reach. Especially in the case of mental illnesses, I don’t think you can–. They need to be in so many places, because I think the number of people affected is incredibly large.”*

(B12)

#### 3.2.5. Future Use of CPGs (Patient Version)

All participants were very interested in CPGs and could *imagine using them* for own purposes in the future. Individuals affected by mental illness as well as family members perceived that CPGs could be useful in handling their own mental illness or that of their relative. The potential benefits of CPG included:

- enhancing consultation with their doctor about therapy options
- education about alternatives to standard treatment options
- information about self-care (e.g., nutrition, exercise, additional psychosocial therapy)
- addresses of additional support options such as psychosocial counseling
- materials for family members and friends for psychoeducation to reduce stigma associated with mental illness.

*“Definitely, because, well, you’ll be informed when you turn up for your consultation. And if you’ve got questions to ask, otherwise you wouldn’t even come up with questions like whether this therapy option would be a possibility. You just wouldn’t know about it. The doctor can’t just recite entire guidelines in a consultation.”*

(B08)

According to the participants, CPGs could be beneficial prior to the onset of symptoms, as well as in an acute phase of mental illness. They could be particularly useful after a new psychiatric diagnosis was made in order to get a first overview, or when personal situations change and new questions arise (e.g., about legal or therapeutic topics or financial support). Participants who were already very well informed and had years of experience with a mental illness assumed that a CPG could no longer offer them much new information, but that it would have been very helpful earlier in their life.

*“Yes, definitely. As I said, all that is not so important to me anymore, now that I know it. I’ve had it for a couple of years now. But in the past it would have been very, very important to me.”*

(B01)

In acute phases of illness, drive and cognitive capacity were not always sufficient to use CPGs. However, the main *barrier to using* a CPG was its lack of accessibility (e.g., not knowing about its existence or not being able to find it). Also, the complexity, scope and incurred costs presented additional barriers.

*“There’s nothing that would hinder me directly, other than that such a long text is naturally cumbersome to deal with and it takes a while to get into it.”*

(B07)

Important *facilitating conditions* mentioned were that CPGs should be easy to access, free of charge and designed to be as easy to use as possible (e.g., not too extensive, and suitable for laypersons).

*“I think it’s good if it’s easily accessible on the internet. That makes it easier, of course. I personally would buy also a book, no doubt about it, but if it’s available free of charge, whether digitally or as a brochure or a book, that makes it easier, of course.”*

(B15)

#### 3.2.6. Specific Needs and Content for Individuals Affected of Mental Illness and Family Members Regarding CPGs (Patient Version)

*Individuals affected by mental illness* emphasized that CPGs should contain a kind of “first aid”- or “first steps”-chapter to be used for orientation after receiving a new diagnosis. Detailed information about psychiatric clinics should be included to destigmatize them and facilitate help seeking when needed. CPGs should be illustrative and include examples of the symptomatology of an illness. An address section with contact information would also be important.

*“I see two clearly different situations: I’m someone at the very beginning and notice that things just aren’t working right now. Everyone wants something from me and I can’t handle it somehow. I feel overwhelmed and I’m having emotional outbursts just like that without knowing why. What is needed at that moment is basic, concise information and first-aid guidance, so that you can feel understood and you’ll have a word for it, or that you’ll know where to turn and you don’t have to just take it or wait any longer. And then there are information leaflets that are meant for people whose situation is more advanced, who perhaps are looking for a therapist or who are somehow deeper in it.”*

(B04)

According to the *family members* interviewed, they emphasized the value of receiving information about the illness and treatment options even in situations where the individuals affected by a mental illness themselves were not able to involve anyone in the process. CPGs could provide them with knowledge to be able to support their family member. These chapters could be identical for individuals affected by mental illness and family members. The following content would be helpful for family members:

- Advice for social interaction with the person affected by mental illness (“dos and don’ts”)
- Information on what you can do together at home in everyday life to reduce symptoms and to favorably influence the course of the illness
- Tips for self-care
- Advice for dealing with feelings of powerlessness and despair
- Encouragement to seek help for oneself when needed.

*“I mean, we’ve reached a dead end with my mother. It would be nice if there were a chapter called ‘How to get out of a dead end’, but I’m being realistic. They are put together in such a way that–. There are many therapies that are being offered, and if the patient is willing to go along with all that, that’s great, but—. Oh, it’s difficult. Anyway, I can’t remember at all if there was a chapter specifically for family members saying ‘do not bury your head in the sand, seek help for yourself before you get burned out on the illnesses of a family member’. That’s also important.”*

(B08)

#### 3.2.7. Updating of Medical Knowledge 

The majority of participants associated progress in medical research with improvements in treatment for themselves and their family members. It was considered essential that updates to treatment recommendations would be transparent.

*“That may be something that concerns me personally. When you see that things are moving forward. You can somehow compare to situation now to seven years ago. Back then, people used to think this and now the view is different. I find it positive to notice that research is progressing and that at some point, when you’re older, you might be able to compare things.”*

(B04)

Nevertheless, rapid gains in knowledge are challenging for non-scientists. Participants perceived study findings and recommendations as contradictory, and the pure amount of information was overwhelming.

*“Very confusing. The thing is, you might read an article today about a study in which it was found that the causes of such and such illness are such and such. Four days later another study comes out that does the exact same thing but with completely opposite results. I think that is the problem and at some point it just gets too much. The sheer volume of it.”*

(B02)

## 4. Discussion

This study indicates that evidence-based patient health information on mental and physical illnesses are used widely, but CPGs and their patient versions are mostly unknown. Not knowing about the existence of CPGs is seen as the greatest barrier to use, consistent with Moreno and Moriana [35] and Correa et al. [34]. Participants expressed a great willingness to use CPGs. The results, therefore, demonstrate the high relevance of promoting CPGs in the general population and of adapting “patient and family member”-versions that are widely available at a low-threshold. Interventions aiming at increasing knowledge about CPGs with educational strategies are recommended as effective implementation strategies in mental health care, including provision of educational materials [42,43,44,45], e.g., in the form of educational workshops for affected individuals and family members [46]. There was a high degree of agreement between the expectations and needs of affected individuals and family members regarding content, design, sources of access, readiness to use, and barriers and facilitators. Only a few specific additional topics were mentioned (e.g., self-care for family members).

### 4.1. Sources of Health Information and CPGs

Sources for accessing health information and CPGs mentioned by participants are mostly consistent with research, with health care providers, medical websites and online search being the main sources [47,48,49,50]. Practitioners are the most frequent source of health information and enjoy the most trust, while internet ranks second in frequency of use, but is significantly less trusted [50]. Participants were critical that evidence-based health information in the form of printed materials was most often available at places where people have already taken the first step and sought help for their mental symptoms. Therefore, an important finding of this study is that health information and access to CPGs should be available in as many places as possible in everyday life and dissemination channels should be comprehensive. 

### 4.2. Target-Group Specific Adaption

In line with research, participants emphasized that health information and CPGs should be adapted as specifically as possible for their successful dissemination [28,35]. Various versions should be developed to accommodate: (a) different target groups (e.g., versions in plain language as well as advanced lay version for people with more prior knowledge), and (2) different phases of an illness and associated limitations (e.g., reduced attention and concentration or lack of drive) and specific needs (e.g., for emergency assistance or for complementary additional therapy options). The study also reflected a need for comprehension-enhancing formal design elements as is consistent with research [23,28,35,51] (e.g., be available in a wide range of formats, be short in length and include audio, visual and interactive elements as well as case studies). As difficulty in interpreting statistical findings was mentioned as a major barrier to understanding evidence-based health information, current recommendations for presenting statistical findings should therefore be followed to enhance comprehensibility (e.g., [28,52,53]). Some patient versions of CPGs that implement these principles already exist (e.g., [54,55,56,57]), but it would be advisable to establish the format of such a complementary patient version as a standard requirement for every CPG.

### 4.3. Trust-Increasing Features

To increase acceptance of evidence-based health information and CPGs, people from the target group should be involved in planning, creation, piloting and evaluation processes, which is also regarded as a key element in research [33,52,58]. Informational materials developed in collaboration with individuals affected by mental illness better meet their specific information needs and are more readable and understandable [28]. The study also shows that trust in evidence-based health information and CPGs can be increased when different points of view are presented (e.g., advantages as well as disadvantages of treatment options) and when they emphasize that current medical knowledge is fluid and can be actualized in the process of gaining scientific knowledge. To accomplish this, recommendations for communicating evidence-based health information should be followed (e.g., [22,59], like reporting the most important benefits and harms, including outcomes for which no evidence was found, appropriate factual and linguistic communication of uncertainties), which is currently not always the case [60]. 

### 4.4. Mental Health Literacy and Empowerment

To improve mental health literacy, Moreno and Moriana [58] suggest providing information and education about causes, symptoms and course of an mental illness as well as treatment options—topics also mentioned by the participants. Also in line with research, the high importance of self-help, peer support and self-management for the recovery process is reflected by the participants [61,62,63]. Therefore, self-help and self-care, as well as coping and management skills, should be promoted as resources in health information and CPGs. The mentioned specific needs by individuals affected by mental illness and family members are consistent with literature (e.g., Flodgren et al. [43]) and should be addressed (e.g., information about how to cope with the pressure of being a carer as well as an encouragement to get help for themselves; for affected individuals, information about first steps after getting an initial diagnosis). 

### 4.5. Specifics of Online Sources of Health Information and CPGs

Although not objectively measured, the participants seem to have a high level of general and mental health literacy (they report being usually good at evaluating health information, have a high educational level and engage with the subject of mental illness for a very long time and in some cases even in self-help associations). Even in this sample with presumably high health literacy, there are difficulties in assessing the quality and reliability of online resources which is consistent with Kim and Xie [64]. Internet sources seem thus generally particularly difficult to assess and apply. On the other side, Brijnath et al. [65] emphasize that usage of web-based interventions are a viable method for improving mental health literacy. So, there is a strong need to improve the ability to apply online health information. Interventions that aim to increase general and mental health literacy should also focus on improving the readability and usability of online health information (e.g., simplicity and clarity in design, understandable language, simplified technical features, interactive features and multimedia formats) [64,65]. Further web platforms for affected people and their family members should offer patient versions of CPGs which are optimized to their needs [66]. All these elements would simultaneously have a positive effect on trust and credibility according to Sbaffi and Rowley [67] and were also mentioned by the participants. They suggested that evidence-based health information and CPGs should be made available by scientific or medical publishers and via trustworthy, impartial and well-known sites (e.g., Federal center for health education, health insurance companies) to further increase trust and facilitate evaluation of the online resources, in line with research [67,68,69,70]. Although mobile apps were viewed rather ambivalently or even negatively by the participants, Kim and Xie [64] recommend providing health information and CPGs via apps and optimizing websites for mobile devices, too, as this would especially support people with low health literacy.

### 4.6. Specifics of Mental Illnesses and the Role of Stigma

Generally, the availability of evidence-based based health information and CPGs was often still perceived as insufficient by affected individuals and their family members, in accordance with research [71,72], and should therefore be improved in the future. Especially for mental illnesses, some participants felt that the availability of information was insufficient compared with physical illnesses, despite perceived improvements in recent years. Stigmatizing perceptions complicated access to adequate information and therefore reduced help-seeking intentions and behavior. Studies also show that lack of knowledge and stigmatizing beliefs about mental illnesses are specific barriers to help-seeking behavior [73,74,75,76]. According to Avdibegović and Hasanović [77], stigma not only reduces help-seeking and receiving help, but also decreases self-esteem, self-efficacy and the belief in one’s own abilities and thereby contributing to social exclusion through discrimination. Participants identified a variety of approaches for improving this situation (e.g., education about mental illness as early as possible, widespread presence of information). Research also shows that interventions early in life (for example school-based campaigns) to educate about mental illness improve mental health literacy in adolescents [78,79,80]. A higher degree of mental health literacy relates to more positive attitudes and less desire for social distance from people with mental illness [81]. Participants also emphasized that health information and CPGs should represent how to live well with mental illness. According to Avdibegović and Hasanović [77], this recovery orientation makes people stronger, gives purpose and meaning in life and leads to social inclusion. Therefore, it should be integrated into evidence-based information and CPGs. 

### 4.7. Strength and Limitations

A strength of this study is the inclusion of individuals affected by mental illness, family members and people who can illuminate both perspectives at once. In some cases, participants’ experience with evidence-based health information on mental illness over the years also allows for the retrospective exploration of changes in the adaption, dissemination and implementation of health information. Furthermore, the sample size was appropriate [82,83], the interviews produced saturated data and study focused on exploring different perspectives rather than on generalizability. A systematic and iterative procedure was used; the coding process and categories were frequently discussed and consented among the authors and quotations were included in Section 3, increasing the confirmability of the study results [41,84]. 

In qualitative research, transferability or generalizability (referring to the external validity of the results), must be critically reviewed. In this study, most participants were already organized in self-help associations and organizations and therefore had extensive knowledge about the topic. It may mean that individuals affected by mental illness and family members who are still at the beginning of dealing with a new diagnosis or who are in an acute phase of the disease might have been missed. As Kim and Xie [64] and Schaeffer et al. [13] found, there is evidence of limited health literacy for people with lower education and income levels, immigrants and men. There may be a sample bias, as men and people with lower formal education levels were underrepresented. Further, there were no people under 30 years old or immigrants in this study. The recruitment methods and the high level of formal education as well as the interviews themselves indicate a rather high mental health literacy, though mental health literacy was not assessed with objective parameters by a valid questionnaire. Thus, the generalizability of the findings may be limited. 

Also, the challenging recruitment process indicated that the considerable commitment of the representatives of individuals affected by mental illness and family members seemed to reach limits regarding their resources. We could observe that interested people responded to the study invitations via newsletter only in small numbers despite expressing strong interest. The study population of individuals affected by mental illness and family members may must be regarded as a group of hard-to-reach population. To address such a study population, a wide range of approved measures should be used in parallel, as described in Röhr et al. [39]. It must be assumed that as interest in participatory research increases, special efforts and resources will need to be provided to encourage and enable individuals affected by mental illness and family members to participate. This challenge must also be met in other health areas by participatory researchers [85]. 

## 5. Conclusions

The study provides information about the specific needs of individuals with mental illnesses and family members regarding evidence-based health information and “patient version” CPGs, as well as tailored adaptation options for this target group and dissemination paths for the psychiatric sector. The results can contribute to optimizing development, adaption, dissemination and implementation of evidence-based health information and CPGs for individuals affected of mental illness and family members. These aspects can contribute to enhancing mental health literacy, better use of mental health services and destigmatization of mental illness.

Important conclusions can be derived for practice: Although there is already a lot of information available about mental illnesses, there is still a need for improvement. Barriers of use can be lowered by adapting health information materials and GPGs to specific target groups and by developing diverse and low-threshold dissemination paths within and outside the health care system. Lay versions additionally to health expert versions of CPGs should be standard practice in the field of mental illness, as well as implementation interventions aimed specifically at those affected by mental illness and their family members. Such interventions should use participative approaches as well as be scientifically evaluated.

Significant research implications also emerged: To increase representativeness and external validity, further exploration of other target groups is recommended: e.g., of individuals from different cultural backgrounds, with lower levels of education, individuals with serious mental illness or immediately after receiving initial diagnosis of mental illness or younger adults. Practical, personal, and structural challenges of participative research should be addressed [85,86]. Individuals who were not already associated with the network of self-help organizations are particularly difficult to reach and hesitate to participate. More recruitment efforts as well as the search for alternative access paths (e.g., via social media or advertising) are needed. A future study should focus on recruiting these underrepresented target groups and individuals who are not organized in self-help associations in order to explore their experiences and needs concerning health information and CPGs.

Patient health information for mental illnesses is already widely used but patient versions of CPGs are little known and therefore rarely used. In order to improve the health literacy of individuals with mental illnesses, information about the benefits and availability of CPGs must be provided more intensively.

## Figures and Tables

**Table 1:** Description of sociodemographic and disease-related characteristics of participants.

	Total	Affected by Mental Illness	Relative of an Individual Affected by Mental Illness	Both Affected and Relative
	15	4	5	6
**Sex**				
Female	12	3	5	4
Male	3	1	0	2
**Age** in years: minimum–maximum	30–74	30–56	36–74	33–67
**Highest school degree**, *n*				
Secondary school diploma	4	2	0	2
High school diploma/ baccalaureate/A-level	11	2	5	4
**Highest professional education**, *n*^1^				
Vocational training	5	2	2	1
Professional school degree	3	2	0	1
University degree	6	0	3	3
**Employment situation ^2^**, *n*				
Full-time employed (≥35 h)	3	0	2	1
Part-time employed (15–34 h)	7	3	1	3
Marginally employed (≤14 h)	2	1	0	1
Parental leave	1	0	1	0
Retired	2	0	1	1
Else ^2^	1	0	0	1
**Mental illness**, *n*				
Unipolar depression	10	4	0	6
Bipolar depression	3	0	2	1
Anxiety disorder	4	2	1	1
Personality disorder	2	1	0	1
Schizophrenia	2	0	2	0
Else ^3^	3	0	1	2
**Duration of illness** in years: minimum–maximum	2–27	2.5–20	3–22	2–27 ^4^

Notes. ^1^ = Multiple answers possible; ^2^ = Lecturer activity; ^3^ = Adjustment disorder, autism, post-traumatic stress disorder and dissociative personality disorder; ^4^ = *N* = 5 (1 missing answer).

## Data Availability

All data generated or analyzed during this study are available from the corresponding author on request.
